# GM-CBAM-ResNet: A Lightweight Deep Learning Network for Diagnosis of COVID-19

**DOI:** 10.3390/jimaging11030076

**Published:** 2025-03-03

**Authors:** Junjiang Zhu, Yihui Zhang, Cheng Ma, Jiaming Wu, Xuchen Wang, Dongdong Kong

**Affiliations:** 1 College of Mechanical and Electrical Engineering, China Jiliang University, Hangzhou 310018, China; zjj602@yeah.net (J.Z.); p22010855036@cjlu.edu.cn (Y.Z.); macheng1802021@163.com (C.M.); 2 School of Mechatronic Engineering and Automation, Shanghai University, Shanghai 200444, China; wjm0712@shu.edu.cn; 3 Department of Electrical and Electronic Engineering, Xi’an Jiaotong-Liverpool University, Suzhou 215123, China; xuchen.wang@xjtlu.edu.cn

**Keywords:** COVID-19, ECG images, ghost module (GM), convolutional block attention module (CBAM), lightweight, deep learning

## Abstract

COVID-19 can cause acute infectious diseases of the respiratory system, and may probably lead to heart damage, which will seriously threaten human health. Electrocardiograms (ECGs) have the advantages of being low cost, non-invasive, and radiation free, and is widely used for evaluating heart health status. In this work, a lightweight deep learning network named GM-CBAM-ResNet is proposed for diagnosing COVID-19 based on ECG images. GM-CBAM-ResNet is constructed by replacing the convolution module with the Ghost module (GM) and adding the convolutional block attention module (CBAM) in the residual module of ResNet. To reveal the superiority of GM-CBAM-ResNet, the other three methods (ResNet, GM-ResNet, and CBAM-ResNet) are also analyzed from the following aspects: model performance, complexity, and interpretability. The model performance is evaluated by using the open ‘ECG Images dataset of Cardiac and COVID-19 Patients’. The complexity is reflected by comparing the number of model parameters. The interpretability is analyzed by utilizing Gradient-weighted Class Activation Mapping (Grad-CAM). Parameter statistics indicate that, on the basis of ResNet19, the number of model parameters of GM-CBAM-ResNet19 is reduced by 45.4%. Experimental results show that, under less model complexity, GM-CBAM-ResNet19 improves the diagnostic accuracy by approximately 5% in comparison with ResNet19. Additionally, the interpretability analysis shows that CBAM can suppress the interference of grid backgrounds and ensure higher diagnostic accuracy under lower model complexity. This work provides a lightweight solution for the rapid and accurate diagnosing of COVD-19 based on ECG images, which holds significant practical deployment value.

## 1. Introduction

In late 2019, novel coronavirus disease (COVID-19) caused by severe acute respiratory syndrome coronavirus (SARS-CoV-2) broke out and developed into a worldwide pandemic [1]. According to the latest real-time statistics of the World Health Organization (WHO), there were 704,753,890 confirmed COVID-19 cases and 7,010,681 cumulative deaths worldwide as of 13 April 2024, 01:00 GMT [2]. COVID-19 has also caused many other issues, such as the rising price of raw materials [3], enterprise closures [4], and massive labor unemployment. The global economic growth rate fluctuated greatly during the COVID-19 pandemic, according to International Monetary Fund (IMF) [5]. In short, COVID-19 has not only caused significant economic impact but also seriously endangered people’s health.

COVID-19 can cause severe damage to the human liver, kidney, and respiratory and nervous systems [6]. Although medical experts are working on viral genome analysis [7] and vaccine development [8] to combat COVID-19, there are still no drugs that can effectively deal with the constantly mutating virus. At present, the early detection of COVID-19 positive patients is the main means to curb the growth of infection cases [9]. Common methods for diagnosing COVID-19 include X-ray [10], reverse transcription-polymerase chain reaction (RT-PCR) [11], and computed tomography (CT). Wang et al. [12,13,14] utilized deep learning algorithms to automatically diagnose COVID-19 from CT images, and its success rate is constantly improving. Recently, some studies have found that COVID-19 can also cause damage to the cardiovascular system of patients [15], such as atrial fibrillation, ST-T abnormalities, acute pericarditis, tachycardia, and myocardial infarction [16,17,18]. Thus, electrocardiograms (ECG) are expected to achieve the early diagnosis of COVID-19 since ECG abnormalities appear earlier than COVID-19 symptoms. Additionally, ECGs have the advantages of being low cost, non-invasive, and radiation free. Naturally, using ECG to diagnose COVID-19 is becoming a relatively new research direction [19,20].

Recently, with the rapid development of artificial intelligence (AI), ECG has been widely used in arrhythmia diagnosis, myocardial ischemia detection, and so on [21,22]. In terms of using ECG to diagnose COVID-19, most studies are carried out based on the ECG database containing COVID-19 patients, which was established by Khan et al. [23]. The ECG image in the database is printed on the recording paper by ECG recorder, and cardiologists observe the waveform changes of ECG signal directly so as to judge the health status of the patient.

Based on the ECG database [23], many studies on using ECG to diagnose COVID-19 have been carried out. Shahin et al. [24] utilized image segmentation, background elimination and data augmentation to obtain more filtered ECG images, and used them to train several convolutional neural network (CNN) models (including VGG16, VGG19, ResNet50, Densenet201, and InceptionV3). It was found that VGG16 achieved the highest accuracy rate of 85.92% in the binary classification of normal and COVID-19. Ozdemir et al. [25] proposed a novel hexaxial feature mapping by utilizing gray-level co-occurrence matrix (GLCM) to extract features from the preprocessed ECG images. The generated hexaxial mapping images are used as the input of a modified AlexNet. Experimental results show that the classification accuracy of the proposed method exceeds 90% (i.e., 96.2% and 93%) in both binary-classification scenarios. Sobahi et al. [26] utilized an attention-based 3D CNN with residual connections (RCs) to detect the preprocessed ECG images. The proposed model achieved average accuracies of 99% and 92% in binary-classification and three-classification tasks, respectively. The above methods [24,25,26] can directly locate or extract the unique features of COVID-19 through preprocessing to achieve better diagnostic accuracy. However, these studies [24,25,26] require image segmentation and preprocessing to remove background, which is somewhat time-consuming for rapid diagnosis.

Another approach is to improve diagnostic accuracy by increasing model complexity, which can avoid image preprocessing. Since deep learning has the advantage of carrying out feature extraction automatically, many scholars are turning to using complete 12-lead ECG images as a whole for training and prediction. Irmak [27] carried out research on COVID-19 diagnosis by utilizing a modified VGG16 under the case of binary-classification, three-classification, and four-classification task, respectively. The overall accuracy of the modified VGG16 reached 98.57%, 93.2%, 96.74%, 86.55%, and 83.05%, respectively. The deficiency is that the number of model parameters reached up to 127.5M, due to the existence of a massive fully connected layer. Attallah [28] presented a feature extraction method by combining the features of the last fully connected layer and the discrete wavelet transform (DWT) of the last average-pooling layer, which come from five CNN models. Subsequently, combined with feature selection and voting classification, the average accuracy of the presented method reaches up to 98.8% and 91.73% in the binary-classification and three-classification tasks, respectively. However, the parameter quantity of the presented method is still relatively large. Rahman et al. [29] compared six CNN models (ResNet18, ResNet50, ResNet101, InceptionV3, DenseNet201 and MobileNetv2) in the binary-classification, three-classification, and five-class classification tasks, respectively. The experimental results show that Densenet201 performs the best in binary- and three-classification (99.1%, and 97.36%), and InceptionV3 outperforms other methods in five-classification (97.83%). Note that Densenet201 and InceptionV3 still have a large number of model parameters, reaching up to 18.1 M and 23.9 M, respectively. It can be found from these studies [27,28,29] that the method with more parameters can achieve better diagnostic accuracy. The deficiency is that complex models are not conducive to actual deployment in clinical settings.

From the previous studies, it can be found that by utilizing unique preprocessing [24,25,26] or increasing model complexity [27,28,29], diagnostic accuracy can be greatly improved. Or in other words, CNN models still can obtain the accurate diagnosing of COVID-19 based on ECG images, even without complex preprocessing. Objectively speaking, increasing of the model parameters is conducive to improving the diagnostic accuracy in multi-classification task that involves more heart diseases. However, parameter quantity directly affects the model complexity, which cannot be infinitely increased. Additionally, complex preprocessing and increasing model complexity are not conducive to actual deployment.In this way, our research objectives on how to achieve rapid and accurate diagnosis COVID-19 based on ECG images have gradually become clear and definite: (1) without complex preprocessing; (2) lightweight model, i.e., relatively low model complexity; and (3) relatively superior diagnostic performance. In summary, we try to explore a technique that can avoid complex preprocessing and high model complexity, and can realize the rapid and accurate diagnosing of COVID-19 based on ECG images.

In this work, a lightweight deep learning network named GM-CBAM-ResNet is presented to make a rapid and accurate diagnosis of COVID-19 for the ECG database containing COVID-19 patients. Inspired by utilizing lightweight models to identify hyper-spectral images with multi-dimensional complex information [30], the Ghost module (GM) is introduced to solve the problem of the high complexity of CNN models. To our knowledge, no previous studies have applied GM to ECG signal processing. Additionally, inspired by utilizing the attention mechanism to suppress background noise interference [31], the convolutional block attention module (CBAM) is added to further improve the diagnostic accuracy. This is due to the consideration that ECG images typically contain substantial grid backgrounds that may interfere with diagnosis. For this reason, CBAM is integrated to ensure focus on the relevant ECG signal features while suppressing grid background noises. Thus, a novel lightweight CNN model is generated by fusing GM and CBAM into a classic CNN model (i.e., ResNet) simultaneously. Additionally, comprehensive analyses are carried out on the four methods, i.e., ResNet, GM-ResNet, CBAM-ResNet, and GM-CBAM-ResNet, so as to fully demonstrate the role of GM and CABM.

In summary, our paper has the following contributions:

(1) A lightweight CNN model is proposed by fusing GM and CBAM into ResNet.
(2) The training time and test time are significantly reduced by introducing GM.
(3) The accuracy in diagnosing COVID-19 is enhanced by utilizing CBAM to suppress the interference of grid background in ECG images.
(4) Interpretability analysis is carried out to illustrate the reliability of GM-CBAM-ResNet.

The effectiveness of the proposed GM-CBAM-ResNet is validated through two cases (i.e., sample balance and sample imbalance). Experimental results show that GM-CBAM-ResNet still can achieve better diagnostic performance, even under less model complexity.

This paper is organized as follows. Section 2 introduces the backgrounds of ResNet, GM, CBAM, and GM-CBAM-ResNet. The performance indicators are described in Section 3. Analysis of the proposed GM-CBAM-ResNet and the experimental results are provided in Section 4. Other details related to GM-CBAM-ResNet and future research focuses are given in Section 5. Finally, Section 6 concludes this paper.

## 2. Methodology

**Figure 1:**
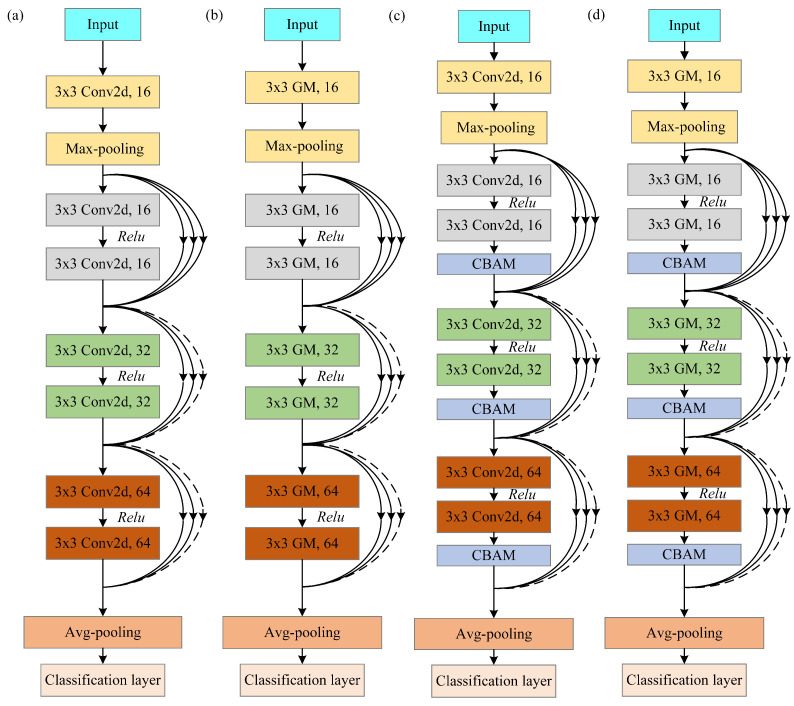
Network structure: (**a**) ResNet19; (**b**) GM-ResNet19; (**c**) CBAM-ResNet19; (**d**) GM-CBAM-ResNet19. GM-ResNet is constructed by replacing the convolution module of ResNet with GM. CBAM-ResNet is constructed by adding CBAM after each residual block of ResNet. GM-CBAM-ResNet is constructed by replacing the convolution module with GM and adding CBAM after each residual block of ResNet.

The construction of GM-CBAM-ResNet is illustrated in Figure 1. The related modules (ResNet, GM, and CBAM) and GM-CBAM-ResNet are introduced as follows:

### 2.1. ResNet

By means of the residual block, ResNet [32] is proposed to eliminate gradient disappearance or gradient explosion, which often occurs in the training process of deep neural network. The detailed operation process of the residual block is provided in Appendix B.

Note that the specific name of the ResNet is determined according to the total number of standard convolution layers, such as ResNet19 and ResNet50.

Due to the wide application of ResNet in medical image processing [33], in this work, the classic ResNet19 is selected as the basic framework as shown in Figure 1a. It consists of one standard convolution layer, one max-pooling layer, nine residual blocks, and one avg-pooling layer.

### 2.2. Ghost Module

To reduce the computational costs required for the standard convolution layer, the Ghost module (GM) [34] is proposed by utilizing deep group convolution to generate more features based on fewer convolution features. The detailed operation process of the GM is provided in Appendix C.

### 2.3. Convolutional Block Attention Module

To enhance the focus on crucial locations and improve the classification performance of CNN without adding a lot of network parameters, the convolutional block attention module (CBAM) [35] is proposed by combining the channel and spatial attention mechanisms. The detailed operation process of GM is provided in Appendix D.

### 2.4. The Novel GM-CBAM-ResNet Model

The standard convolution module makes ResNet have a large number of model parameters, which has high requirements for device memory [36].

In this work, GM [34] is used to replace the standard convolution module in the residual block of ResNet so as to reduce the number of convolutional filters (i.e., model parameters) in ResNet. The obtained GM-ResNet19 is shown in Figure 1b. The specific structure of GM-ResNet19 includes a GM, a max-pooling module, nine residual blocks consisting of GM, and an avg-pooling module.

To enhance the ability of feature attention, CBAM [35] is added after each residual block of ResNet so as to help the network focus on crucial features and suppress noises. The obtained CBAM-ResNet19 is shown in Figure 1c. CBAM-ResNet19 includes a convolution module, a max-pooling module, nine residual blocks including CBAM, and an avg-pooling module.

In order to focus on more crucial location information so as to improve the classification rate of ECG images, this work proposes a more advanced model by integrating both GM and CBAM into ResNet. This combines the advantages of both GM and CBAM, which is conducive to improving the classification rate of ECG images. The obtained GM-CBAM-ResNet19 is shown in Figure 1d.

## 3. Performance Indicators

In this work, except for multi-classification accuracy, another three performance indicators (*Sensitivity*, *Specificity*, and *F1-score*) for binary classification are also adopted to further evaluate the diagnostic performance of the proposed GM-CBAM-ResNet:

(1) $$\begin{array}{c} Accuracy=\frac{\mathrm{Correctly}\mathrm{classified}\mathrm{samples}}{\mathrm{Total}\mathrm{sample}\mathrm{size}} \end{array}$$

(2) $$Sensitivity=\frac{TP}{TP+FN}$$

(3) $$Specificity=\frac{TN}{TN+FP}$$

(4) $$F1\text{-}Score=\frac{2*Precision*Recall}{Precision+Recall}=\frac{2*TP}{2*TP+FP+FN}$$

where *TP* represents predicting a positive sample as a positive sample, *TN* represents predicting a negative sample as a negative sample, *FP* represents predicting a negative sample as a positive sample, and *FN* represents predicting a positive sample as a negative sample. Precision=TP/(TP+FP), Recall=TP/(TP+FN).

Sensitivity and Specificity represent the ability to predict positive cases and negative cases, respectively. Recall is equal to Sensitivity and focuses on the missed detection of positive cases. Precision is contrary to Recall and focuses on the false detection of positive cases. F1-Score is the harmonic mean of Precision and Recall, which is closer to the smaller value of Precision and Recall. This measurement is more rigorous.

The training time and test time are also adopted to analyze the feasibility of the proposed GM-CBAM-ResNet. These two indicators have a significant influence on model development, model efficiency, and model cost.

## 4. Experimental Results and Analysis

In order to solve the problem that simple lightweight models cannot achieve high diagnostic accuracy on ECG images, a new lightweight neural network named GM-CBAM-ResNet is proposed by combining ResNet, the Ghost module (GM), and the convolutional block attention module (CBAM). The feasibility of GM-CBAM-ResNet is analyzed from three aspects: model performance, model complexity, and model interpretability.

### 4.1. Experimental Setup

As for hardware, all experiments are carried out based on CPU i9-10900X, RAM 64 GB, and two graphics cards (RTX3060 12 GB). As for software, all neural network models are constructed by using Numpy 1.21 and PyTorch 1.10 installed on Ubuntu 20.04.

### 4.2. Data Source and Preprocessing

This work is carried out based on a publicly available dataset, i.e., the open ‘ECG Images dataset of Cardiac and COVID-19 Patients’ [23]. This ECG dataset is created under the auspices of Ch. Pervaiz Elahi Institute of Cardiology Multan, Pakistan. The collected ECG images are reviewed by medical professors through a remote ECG diagnosis system, under the supervision of senior medical professionals with experience in ECG interpretation. This guarantees the reliability and correctness of the obtained ECG dataset. It includes 250 images of COVID-19 patients, 77 images of myocardial infarction (MI), 548 images of abnormal heart beats, 203 images of individuals with a history of MI, and 859 images of healthy individuals.

**Figure 2:**
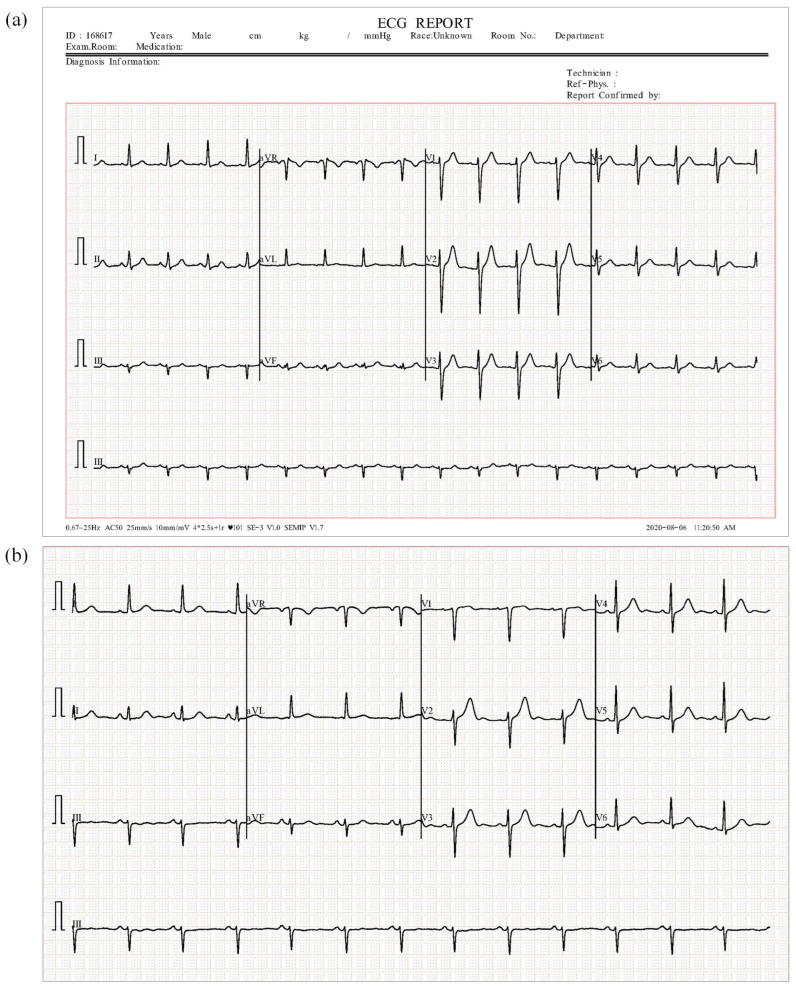
ECG images of COVID-19: (**a**) an original ECG image; (**b**) the ECG image to be preprocessed. The original ECG image contains auxiliary recording information, such as patient ID, report generation time, and diagnostic information. In the preprocessed ECG image, the auxiliary recording information is removed to retain only the 12-lead ECG signals.

Only the 12-lead ECG in the ECG image is preserved in the modeling process so as to remove the interference from auxiliary recording information, such as patient ID and report generation time. The retained image content is illustrated in Figure 2. Moreover, all the images are reshaped and resized to 224×224 so as to avoid the influence of an inconsistent size.

In the process of model construction and evaluation (i.e., training and test), the images (RGB, pixel size: 224×224×3) to be preprocessed are converted to gray-level images and then copied three times to create new images (similar to RGB). The reconstructed images are adopted as the input. As for each image, the corresponding output is a one-hot vector that contains three elements. Each element can be considered the probability of the corresponding category. The relationship between the label and one-hot vector for each category is listed in Table 1.

### 4.3. Model Construction

In this work, the proposed GM-CBAM-ResNet is utilized for the diagnosis of COVID-19 based on ECG images. The overall structure of the proposed GM-CBAM-ResNet is shown in Figure 1d. GM-CBAM-ResNet is constructed by combining ResNet, GM, and CBAM. Firstly, the standard convolution module in the residual block of ResNet is replaced by GM. Secondly, CBAM is added in each residual block of GM-ResNet. The detailed network structure and model parameter information of ResNet, GM-ResNet, CBAM-ResNet, and GM-CBAM-ResNet are provided in Appendix F.

The network structure of ResNet19 is shown in Table A1. It can be found that in the module ‘Residual_dashed’, the sizes of the input and output are kept consistent according to Equations (A2) and (A3), under the setting of [p s] = [1 1]. However, in the module ‘Residual_dashed’, the height and width of the input are reduced by half, under the different settings of [p s] in each layer ([1 2], [1 1], [0 2]). Note that in the construction of GM-ResNet, CBAM-ResNet and GM-CBAM-ResNet, the sizes of the input and output in each residual block should be the same as those in ResNet. Based on this guideline, the specific settings of the hyper-parameters in GM-ResNet, CBAM-ResNet, and GM-CBAM-ResNet are summarized in Table 2.

As for GM-ResNet, the convolution module of ResNet is replaced by GM. In GM, two types of convolution kernel are needed, i.e., the standard convolution kernels $f^{m\times k\times k\times c_{in}}$ and the *m* group convolution kernels $m\times f^{d\times d\times (n-1)}$ as mentioned in Appendix C. To obey the above guideline, the size of the group convolution kernel in GM is set to d=k=3. The ‘mapping ratio’ of GM is set to λ=1/n=0.5. Thus, n=2 and $m=c_{\mathrm{out}}/2$ are obtained, as recommended in [34]. Based on these parameter settings, GM-ResNet can be constructed. The network structure of GM-ResNet19 is shown in Table A2.

As for CBAM-ResNet, CBAM is added after each residual block of ResNet. CBAM contains three types of convolution kernel as mentioned in Appendix D. The evolution of avg-pooling and max-pooling in the channel attention module contains two convolution kernels, respectively, i.e., the first $f^{c_{\mathrm{middle}}\times 1\times 1\times c_{\mathrm{in}}}$ and the second $f^{c_{\mathrm{in}}\times 1\times 1\times c_{\mathrm{middle}}}$. The spatial attention module contains one convolution kernel, i.e., $f^{1\times 7\times 7\times 2}$. In this work, the ‘compression ratio’ of the channel attention module is set to $r=c_{\mathrm{in}}/c_{\mathrm{middle}}=8$ as recommended in [35]. $c_{\mathrm{in}}$ is determined by the dimension of the output from the previous layer. When $c_{\mathrm{in}}$ and *r* are determined, $c_{\mathrm{middle}}$ can be obtained. Based on these parameter settings, CBAM-ResNet can be constructed. The network structure of CBAM-ResNet19 is shown in Table A3.

As for GM-CBAM-ResNet, the convolution module is replaced by GM, and CBAM is added after each residual block of ResNet. The ‘mapping ratio’ of GM and the ‘compression ratio’ of CBAM is the same as that in GM-ResNet and CBAM-ResNet, respectively. Based on these parameter settings, GM-CBAM-ResNet can be constructed. The network structure of GM-CBAM-ResNet19 is shown in Table A4.

### 4.4. Performance Evaluation

To reveal the advantages of the proposed GM-CBAM-ResNet, multiple classification tasks are carried out in this work. Model evaluation is carried out under different conditions, i.e., sample balance and sample imbalance, so as to fully validate the effectiveness of the proposed GM-CBAM-ResNet.

The two cases (i.e., sample balance and sample imbalance) correspond to two datasets. The number of samples in each category under the two cases is shown in Table 3. All data samples are gathered from the publicly available ECG dataset as mentioned in Section 4.2. In the case of sample balance, a total of 250 ECG images for each category are selected. In the case of sample imbalance, the whole ECG images for each category are selected.

In this section, ResNet, GM-ResNet, CBAM-ResNet, and GM-CBAM-ResNet are all evaluated. The process of model construction and evaluation is the same for the four methods, which is introduced as follows. For each case (i.e., sample balance and sample imbalance), the corresponding dataset is divided into two subsets, the training dataset and test dataset, which account for 80% and 20%, respectively. Next, the training dataset is used for training four diagnostic models, i.e., determining the model parameters of filters (i.e., convolution kernels) in each method. Then, the test dataset is used to evaluate the diagnostic performance of the constructed models.

The training parameter settings are listed in Table 4. In neural networks, the output of the final layer (i.e., fully connected layer) is the predicted label. In this work, the cross-entropy loss function [37] is used to quantify the difference between the real and predicted labels. Then, the partial derivative (i.e., gradient) of the function loss with regard to the model parameters of each layer is calculated and used for model updating. Constantly updated model parameters can make the function loss smaller and smaller. The initial learning rate is set to 0.001, and the ‘Adam’ optimizer is utilized to optimize the model parameters. This helps to ensure the stability of the training process. To avoid running out of memory, the training dataset is input in mini-batches and participates in training. The size of the mini-batch is set to 32. One epoch refers to one cycle, i.e., all mini-batches have already participated in the training. Iteration stop conditions contain three cases: (1) epoch patience, (2) function loss, and (3) epoch. The threshold of the function loss is set to 0.0001. The epoch is set to 50. When any one of the three conditions is satisfied, the iteration process will stop. This helps to ensure that the model parameters can reach stable values.

**Figure 3:**
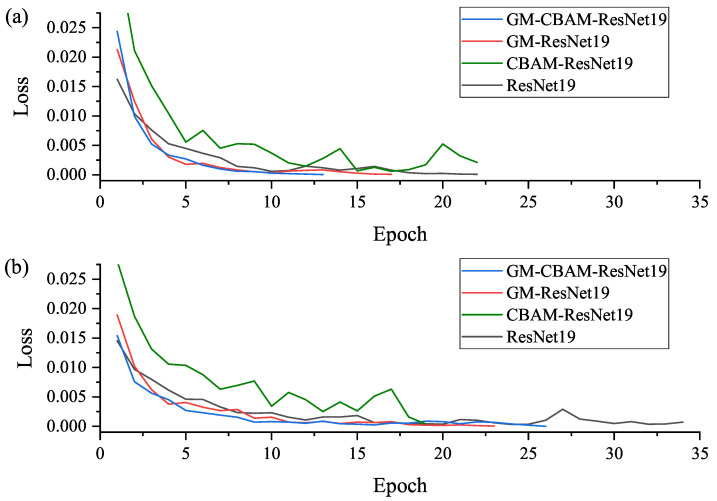
Loss curves of training process: (**a**) loss curves under the case of sample balance; (**b**) loss curves under the case of sample imbalance.

**Figure 4:**
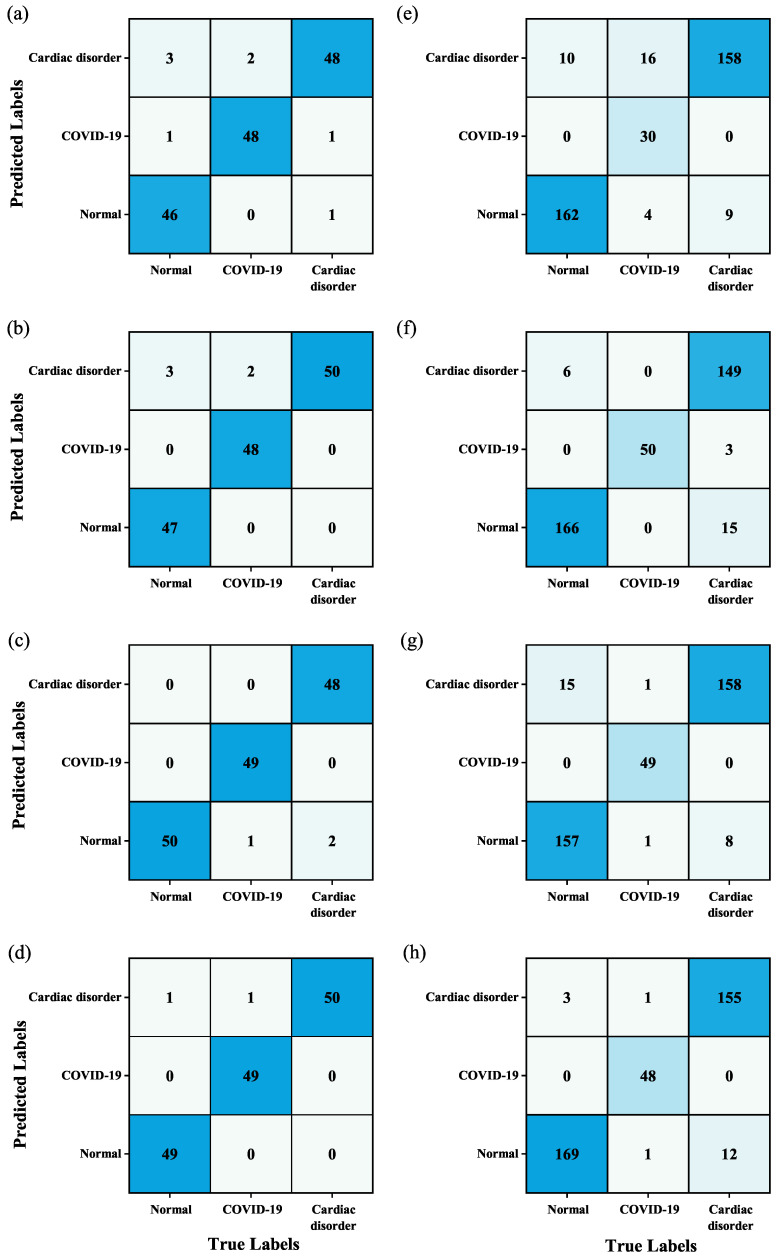
Confusion matrix for the four diagnostic methods under the case of sample balance and sample imbalance: (**a**) ResNet19 under sample balance; (**b**) GM-ResNet19 under sample balance; (**c**) CBAM-ResNet19 under sample balance; (**d**) GM-CBAM-ResNet19 under sample balance; (**e**) ResNet19 under sample imbalance; (**f**) GM-ResNet19 under sample imbalance; (**g**) CBAM-ResNet19 under sample imbalance; and (**h**) GM-CBAM-ResNet19 under sample imbalance.

The training process can be displayed by the change in loss function as shown in Figure 3. The diagnostic model is finally determined when the training process is finished and can be used to predict the categories of test datasets. The experimental results of the two cases (i.e., sample balance and sample imbalance) are listed in Table 5. It can be found that the proposed GM-CBAM-ResNet performs the best in the diagnosis of ECG images. The detailed diagnostic information (i.e., confusion matrix) is shown in Figure 4. It can be found that, after adding GM and CBAM, the misdiagnosis is greatly reduced, especially for COVID-19 under the case of sample imbalance. This phenomenon is particularly close to clinical situations. Thus, GM-CBAM-ResNet is highly recommended for the diagnosis of COVID-19.

### 4.5. Complexity Evaluation

Model complexity directly affects the training and application of the constructed model. In this work, the complexity of the proposed GM-CBAM-ResNet is verified by using the total number of model parameters. In the same hardware environment and computing (programming) approach, fewer model parameters means less computational burden, i.e., less training time and test time.

The total number of model parameters, training time, and test time of the four methods (i.e., ResNet, GM-ResNet, CBAM-ResNet, and GM-CBAM-ResNet) are listed in Table 6, respectively. It can be found that the number of model parameters in GM-ResNet and GM-CBAM-ResNet is only about half of that of ResNet. The training time and test time of GM-ResNet and GM-CBAM-ResNet are also far less than those of ResNet. A high diagnostic rate fully guarantees the feasibility of GM-CBAM-ResNet as a classifier for ECG images. Meanwhile, considering the limited testing time, this ensures the feasibility of GM-CBAM-ResNet in application.

In summary, the model complexity of GM-CBAM-ResNet is significantly reduced since the standard convolution module in the residual block of ResNet is replaced by GM.

### 4.6. Model Interpretability

In the processing and analyzing of medical image data, how to make the diagnostic results of ‘black box’ model interpretable is the focus of researchers’ attention.

In this section, Grad-CAM [38] is utilized to analyze why GM and CBAM can improve the classification performance of the deep neural network. The detailed operation process of Grad-CAM is provided in Appendix E.

**Figure 5:**
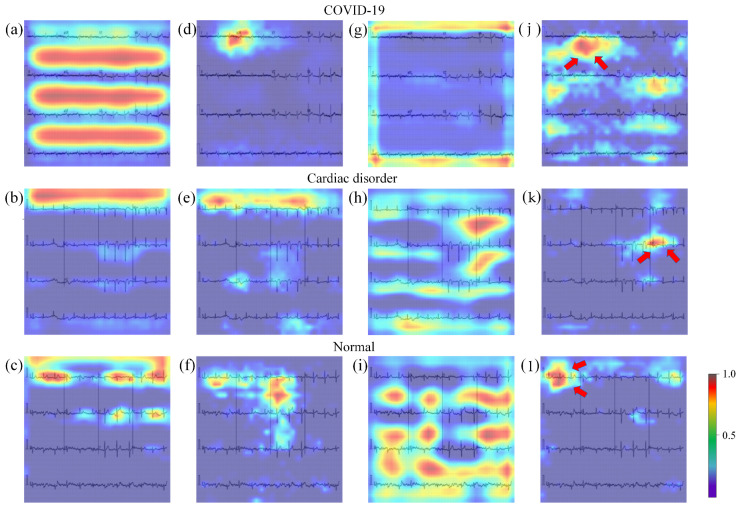
Visualization of ECG images of COVID-19, cardiac disorder, and normal by utilizing Grad-CAM on the corresponding trained deep leaning model: (**a**–**c**) ECG heat maps of ResNet19; (**d**–**f**) ECG heat maps of GM-ResNet19; (**g**–**i**) ECG heat maps of CBAM-ResNet19; (**j**–**l**) ECG heat maps of GM-CBAM-ResNet19.

To obtain the corresponding color ECG thermal map, the last residual block of ResNet, GM-ResNet, CBAM-ResNet, and GM-CBAM-ResNet is connected to Grad-CAM, respectively. The visualized results of the four methods are shown in Figure 5. In the color ECG thermal map, different colors indicate the degree of attention that the model pays to the region. On the other hand, a darker color (red) means greater contribution of the region to the network prediction.

The comparison of Figure 5a–c and Figure 5d–f shows that, compared with ResNet, GM-ResNet focuses significantly less on non-critical areas, indicating that GM improves model accuracy by increasing the proportion of important information. By comparing Figure 5d–f and Figure 5g–i, it can be found that after adding CBAM, GM-CBAM-ResNet is able to locate important ECG leads more accurately. Notably, this effect is more obvious in the ECGs of patients with cardiac disorder. Comparison results show that GM-CBAM-ResNet more easily locates abnormal ECG leads, which is conducive to improving the diagnostic accuracy.

In summary, GM-CBAM-ResNet can focus on the ECG signals as much as possible, rather than the grid background. CBAM helps to locate important ECG leads more accurately.

### 4.7. Comparison with Other Methods

To further reveal the advantages of the proposed GM-CBAM-ResNet, some other classical networks [32,39,40,41,42,43,44,45] for deep learning are also utilized to realize the diagnosis of ECG images.

In this section, the same datasets as shown in Table 3 are used to verify the classification performance of the comparison methods. The training parameter settings of these methods are the same as those in Table 4. The classification performance, model complexity, training time, and test time of each method are listed in Table 7, respectively. It can be found that the proposed GM-CBAM-ResNet performs the best, even in the case of low model complexity. The test time of the proposed GM-CBAM-ResNet is far less than other state-of-the-art methods.

In this work, the accurate detection of COVID-19 is an important research goal. To further test the performance of GM-CBAM-ResNet in the detection of COVID-19, another three performance indicators (sensitivity, specificity, and F1-score) for binary classification as mentioned in Section 3 are evaluated, respectively. COVID-19 is considered a positive sample, while others are set as negative sample. The obtained test results of each method are listed in Table 8. It can be found that, except sensitivity, GM-CBAM-ResNet is superior or comparable to other classical methods. This further shows the superiority of GM-CBAM-ResNet in diagnosing COVID-19.

**Figure 6:**
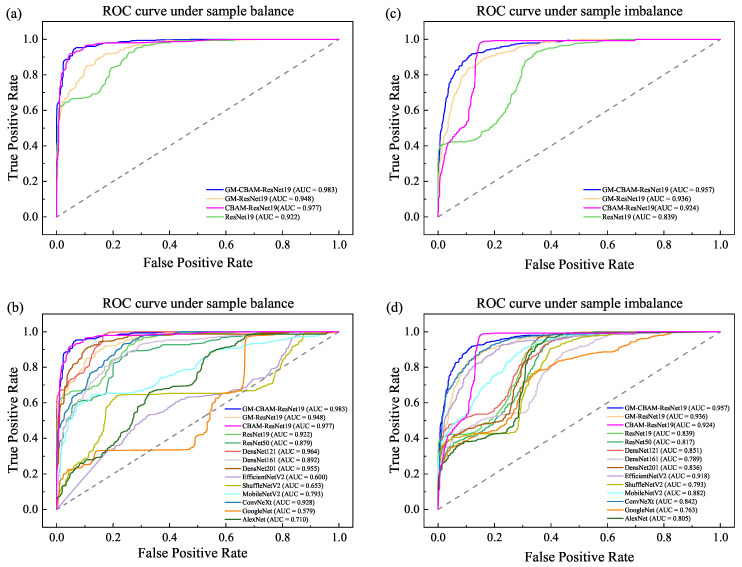
The ROC and AUC of each method.

To validate the generality of GM-CBAM-ResNet for various situations, the receiver operating characteristic (ROC) curve and area under curve (AUC) of each method are provided in Figure 6. The three categories (i.e., COVID-19, cardiac disorder, and normal) as shown in Table 3 are considered positive sample, respectively, to test the performance of each method. The average true positive rate and false positive rate of the three tests are adopted. It can be found that the AUC of GM-CBAM-ResNet clearly performs better than GM-ResNet and ResNet, and outperforms other state-of-the-art methods.

In summary, the proposed GM-CBAM-ResNet is especially suitable for the rapid and accurate diagnosing of ECG images.

### 4.8. Model Test on Other Dataset

To verify the generality of the proposed GM-CBAM-ResNet, a chest X-ray (CXR) database that contains COVID-19 is adopted to test its classification performance.

COVID-QU-Ex [46] is a database that is used to detect COVID-19 according to CXR images. It contains a total of 33,920 CXR images, of which 10,701 are normal, 11,956 are COVID-19, and 11,263 are non-COVID infections (viral or bacterial pneumonia). All images are grayscale with a resolution of 256×256.

In this section, data preprocessing is basically the same as that mentioned in Section 4.2. All images are firstly reshaped to 224×224, and then copied three times to create new images (similar to RGB). The reconstructed new images are adopted to evaluate each model as shown in Table 7. As for the partition of the dataset, the same operation as that mentioned in Section 4.4 is carried out. The new images are divided into two subsets, the training dataset and test dataset, which account for 80% and 20%, respectively. The training parameter settings for each method are the same as those in Table 4, which is also mentioned in Section 4.7.

The evaluation result of each method is listed in Table 9. It can be found that the proposed GM-CBAM-ResNet still performs the best, and its test time is relatively shorter. This shows that the proposed GM-CBAM-ResNet has a certain degree of generality. Thus, it can be concluded that, even under different experimental settings, the proposed GM-CBAM-ResNet still can achieve the fast and accurate diagnosis of COVID-19.

## 5. Discussion

Objectively speaking, the development of deep learning in algorithms is mainly reflected in two aspects: (1) proposing new modules; and (2) exploring the combinations of different modules. Finding the optimal combination to achieve better diagnostic results is also a research topic for many scholars.

In this work, a lightweight deep learning network, i.e., GM-CBAM-ResNet, is proposed for the diagnosis of COVID-19. To verify the performance of each module (i.e., GM and CBAM), four methods (i.e., ResNet, GM-ResNet, CBAM-ResNet, and GM-CBAM-ResNet) are evaluated under two cases (i.e., sample balance and sample imbalance), respectively. GM significantly reduces model complexity while maintaining feature representation capabilities. CBAM enhances feature attention by effectively suppressing grid background interference. In theory, the adding of GM and CBAM will result in a lightweight and accurate diagnostic model, which is conducive to improving the classification accuracy. Experimental results show that, under the case of no complex preprocessing, GM-CBAM-ResNet demonstrates its unique advantages in diagnostic accuracy, model complexity, ROC, and AUC.

In ECG diagnosis, the incidence of specific diseases, such as myocardial infarction (MI), is very low. Or in other words, clinical data acquisition is challenging, especially for negative cases. Thus, normal ECG accounts for the majority, which leads to category imbalance [47]. The ECG database [23] used in this study reflects this clinical reality, which contains 250 images of COVID-19, 77 images of MI, 548 images of abnormal heart beats, 203 images of individuals with history of MI, and 859 images of healthy individuals. This is why sample imbalance is also a key research object. As for model performance degradation on sample imbalance, there is currently limited publicly available data on COVID-19 related ECG images. In the future, we will further test model performance on larger datasets.

Grad-CAM is utilized to explain the internal mechanism of deep learning models for accurately classifying ECG images. It can be found that, after adding CBAM, the attention to grid information in the background of ECG images is significantly reduced. It can be seen from Figure 5k that the attention mechanism focuses on the Channel V5, which shows significant inversion in the T-wave. This is one of the crucial diagnostic criteria adopted by clinicians for myocardial infarction assessment. Moreover, Channel V2∼V4 shows slight elevation in the ST-segment, which accompanied by abnormal Q-waves. It can be seen from Figure 5j that Channel I, -II, -III, and aVF display T-wave abnormalities, respectively. This is one of the specific ECG manifestations in COVID-19 patients. The attention mechanism also focuses on the corresponding regions and the adjacent areas. Objectively speaking, this phenomenon is consistent with the way that clinical doctors diagnose by the changes in ECG voltage. This indicates that GM-CBAM-ResNet can help doctors establish efficient and accurate decision-making.

In this work, the GM-CBAM-ResNet19 model consists of a total of 147,794 parameters. When an imbalanced dataset is adopted, it takes 203 s for training and 0.13 s for testing. Clinical computers typically have configurations that are less powerful than the experimental environment, which may lead to an increase in inference time. However, the significant reduction in model parameters makes it more likely for clinical application. The lightweight nature and high diagnostic accuracy of GM-CBAM-ResNet, combined with its low hardware requirements, make it particularly suitable for various medical scenarios, such as wearable devices and mobile medical diagnostics.

Note that there are some limitations in the current work. While the attention mechanism demonstrates promising results, it does not completely align with the focus areas of clinicians. This is somewhat evident in Figure 5j. Although some attention is concentrated on the lead positions, there is still a large amount of attention distributed outside the lead areas. In the future work, improving the attention mechanism so as to better match clinical expertise and enhance algorithmic interpretability remains an important direction. The current work has not yet undergone specific noise tolerance testing due to the limitations in the dataset scope. Thus, the robustness and generalizability of the algorithm require further validation and improvement.

## 6. Conclusions

In this work, a lightweight deep learning network named GM-CBAM-ResNet is proposed for the diagnosis of COVID-19 according to ECG images. GM-CBAM-ResNet is generated by replacing the convolution operation in each residual module of ResNet with GM and adding a CBAM in each new residual module. GM greatly reduces the model complexity of ResNet. The interpretability analysis of GM-CBAM-ResNet is attempted to carry out and find that CBAM can suppress the interference of grid backgrounds in ECG images. Experimental results show that GM-CBAM-ResNet performs better than ResNet, GM-ResNet, and CBAM-ResNet in terms of accuracy and efficiency. Moreover, the comparison results show that GM-CBAM-ResNet also performs better than other state-of-the-art methods.

Two types of datasets that contain COVID-19 fully validate the effectiveness of the proposed GM-CBAM-ResNet. This paper provides theoretical guidance for the accurate and effective diagnosis of COVID-19 in clinical practice.

In the future, we will carry out some clinical trials to test the diagnostic performance and time consumption of GM-CBAM-ResNet in real clinical scenarios. If feasible, we will transplant it to mobile devices. We will also investigate the diagnostic effectiveness of GM-CBAM-ResNet on other medical signals, such as X-rays and microscopy images.

## Figures and Tables

**Table 1:** Relationship between the label and one-hot vector for each category.

Category	Label	One-Hot Vector
COVID-19	0	[1,0,0]
Cardiac disorder	1	[0,1,0]
Normal	2	[0,0,1]

**Table 2:** Hyper-parameter settings in GM-ResNet, CBAM-ResNet, and GM-CBAM-ResNet.

Module	Hyper-Parameters
GM	d=3;λ=0.5
CBAM	r=8

Note: $r=c_{\mathrm{in}}/c_{\mathrm{middle}}$ is the ‘compression ratio’ of the channel attention module as mentioned in Appendix D.

**Table 3:** Dataset acquisition for sample balance and sample imbalance.

Case	Dataset	COVID-19	Cardiac Disorder	Normal
Sample balance	Dataset 1	250	250	250
Sample imbalance	Dataset 2	250	828	859

Note: Cardiac disorder consists of myocardial infarction (MI), abnormal heart beats and history of MI.

**Table 4:** The training parameter settings.

Learning Rate	Optimizer	Batch Size	Epoch Patience	Function Loss	Epochs
0.001	Adam	32	10	0.0001	50

Note: ‘Epoch patience’ refers to the training process will stop when the lowest loss value of the cross-entropy loss function on the training dataset persists for ten consecutive rounds with no changes.

**Table 5:** Classification accuracy of the four diagnostic methods.

	ResNet19	GM-ResNet19	CBAM-ResNet19	GM-CBAM-ResNet19
Sample balance	94.7	96.7	98	98.7
Sample imbalance	90	93.8	93.8	95.6

**Table 6:** The total number of model parameters, training time, and test time of the four diagnostic methods.

Method	Total_num_params	Training Time (s)	Test Time (s)
GM-CBAM-ResNet19	147,794	57/203	0.05/0.13
GM-ResNet19	138,848	44/185	0.04/0.11
CBAM-ResNet19	275,362	78/270	0.08/0.24
ResNet19	270,448	76/262	0.08/0.23

Note: ‘Total_num_params’ refers to the total number of model parameters. As for the training time and test time, value1/value2 correspond to the case of sample balance and sample imbalance, respectively.

**Table 7:** Comparison of the proposed GM-CBAM-ResNet and other state-of-the-art methods.

Method	Accuracy (%)	Total_num_params (M)	Training Time (s)	Test Time (s)
GM-CBAM-ResNet19	98.7/95.6	0.15	57/203	0.05/0.13
GM-ResNet19	96.7/93.8	0.14	44/185	0.04/0.11
CBAM-ResNet19	98/93.8	0.28	78/270	0.08/0.24
ResNet19 [32]	94.7/90	0.27	76/262	0.08/0.23
ResNet50 [32]	96.7/93.8	23.5	245/928	0.33/0.89
DensNet121 [39]	97.3/94.3	6.95	111/445	0.18/0.5
DensNet161 [39]	94.7/92.3	26.47	313/1181	0.41/1.36
DensNet201 [39]	95.3/93.1	18.1	190/817	0.29/0.71
EfficientNetV2 [40]	94.7/93.6	4.01	96/311	0.08/0.2
ShuffleNetV2 [41]	92.7/91.5	1.25	71/196	0.06/0.16
MobileNetV2 [42]	88/86.4	2.23	90/232	0.06/0.17
ConvNeXt [43]	94/93.3	87.57	525/1455	1.14/2.95
GoogleNet [44]	86.7/85.6	10.31	132/660	0.21/0.54
AlexNet [45]	84.7/82.3	14.59	136/689	0.24/0.6

Note: ‘Total_num_params’ refers to the total number of model parameters. As for the number of model parameters, $1M=10^{6}$. As for the accuracy, training time, and test time, value1/value2 correspond to the case of sample balance and sample imbalance, respectively.

**Table 8:** Comparison of identification performance of various methods for binary classification.

Method	Sensitivity (%)	Specificity (%)	F1-Score (%)
GM-CBAM-ResNet19	98/96	100/100	99/98
GM-ResNet19	96/100	100/99.1	98/97.1
CBAM-ResNet19	98/98	100/100	99/99
ResNet19 [32]	96/60	98/100	96/75
ResNet50 [32]	96/94	100/100	98/96.9
DensNet121 [39]	96/98	100/98.8	98/95.1
DensNet161 [39]	94/96	100/98.2	96.9/92.3
DensNet201 [39]	96/98	97/98.2	95/93.3
EfficientNetV2 [40]	92/96	100/98.2	95.8/92.3
ShuffleNetV2 [41]	96/96	100/97.1	98/88.9
MobileNetV2 [42]	100/96	85/92	87/86.9
ConvNeXt [43]	95/96	100/98.5	97.4/93.2
GoogleNet [44]	82/92	100/100	90.1/95.8
AlexNet [45]	94/96	88/89	86.2/87.9

Note: As for the Sensitivity, Specificity and F1-score, value1/value2 corresponds to the case of sample balance and sample imbalance, respectively.

**Table 9:** Model test on the chest X-ray (CXR) database [46].

Model	Accuracy (%)	Total_num_params (M)	Training Time (s)	Test Time (s)
GM-CBAM-ResNet19	94.3	0.15	1313	0.67
GM-ResNet19	92.2	0.14	1087	0.56
CBAM-ResNet19	90.7	0.28	2035	0.93
ResNet19 [32]	91	0.27	1994	0.87
ResNet50 [32]	91.2	23.45	18,332	5.58
DensNet121 [39]	92.3	6.87	8345	2.77
DensNet161 [39]	91.7	26.25	18,041	5.43
DensNet201 [39]	92.6	17.86	14,418	4.47
EfficientNetV2 [40]	92.2	4.1	4672	1.8
ShuffleNetV2 [41]	90.6	1.21	1850	1.19
MobileNetV2 [42]	87	2.19	3525	1.43
ConvNeXt [43]	86.8	87.54	30,525	12.13
GoogleNet [44]	85.2	10.29	12,634	3.34
AlexNet [45]	82.7	14.58	12,090	4.28

Note: ‘Total_num_params’ refers to the total number of model parameters. As for the number of model parameters, $1M=10^{6}$.

**Figure A1:**
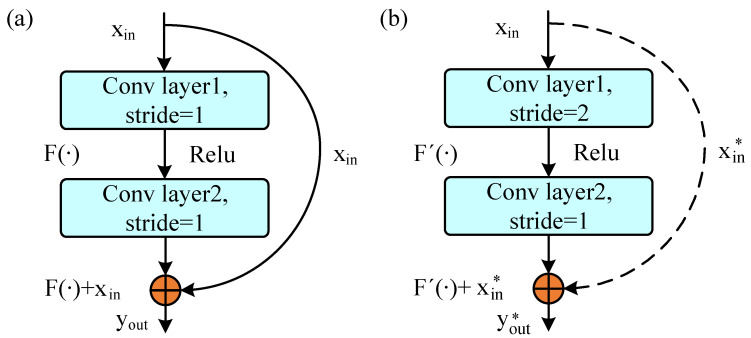
The residual block: (**a**) The ‘solid line’ indicates that the input and output have the same number of channels; (**b**) the ‘dashed line’ indicates that the input and output have different numbers of channels. Note: The ‘dashed line’ refers to the fact that $x_{\mathrm{in}}$ is convolved with the $c_{\mathrm{out}}^{2}$ convolution kernels (with the size of $1\times 1\times c_{\mathrm{in}}^{1}$).

**Figure A2:**
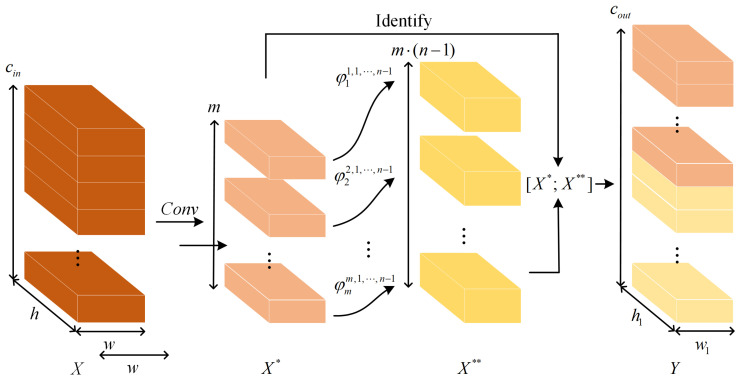
The operation process of the Ghost module (GM).

**Figure A3:**
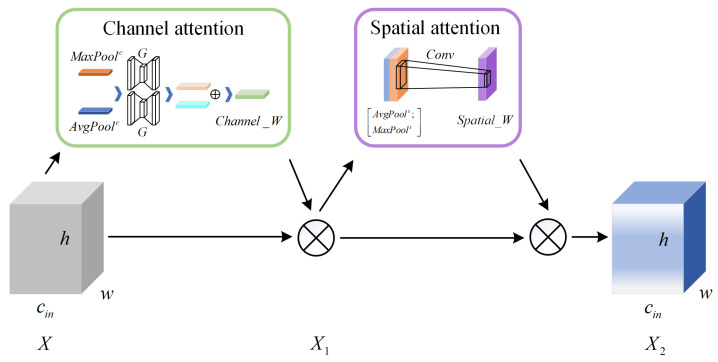
Schematic diagram of the convolutional block attention module (CBAM).

**Figure A4:**
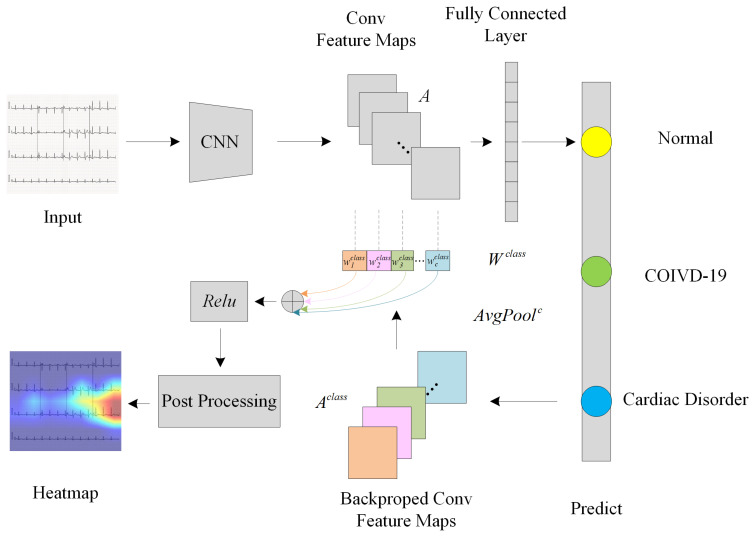
Implementation of Grad-CAM.

## Data Availability

The data used to support the findings of this study are available from the corresponding author upon request.

## Appendix A. Standard Convolution Module

Supposing that $X_{h\times w\times c_{in}}$ represents the input data or image, $Y_{h^{*}\times w^{*}\times c_{out}}$ is the output. The standard convolution operation [48] can be expressed by

(A1) $$Y_{h^{*}\times w^{*}\times c_{out}}=\mathrm{Conv}\left(X_{h\times w\times c_{in}},f^{c_{out}\times k\times k\times c_{in}}\right)$$

(A2) $$h^{*}=\mathrm{fix}\left(\frac{h+2\times padding-k}{stride}\right)+1$$

(A3) $$w^{*}=\mathrm{fix}\left(\frac{w+2\times padding-k}{stride}\right)+1$$

where Conv(·) refers to the standard convolution function. *h* and *w* are the height and width of the input, respectively. $h^{*}$ and $w^{*}$ are the height and width of the output, respectively. $c_{\mathrm{in}}$ and $c_{\mathrm{out}}$ are the number of input channels and output channels, respectively. $f^{c_{\mathrm{out}}\times k\times k\times c_{\mathrm{in}}}$ refers to the $c_{\mathrm{out}}$ $k\times k\times c_{\mathrm{in}}$. fix(·) rounds the processed element to the nearest integer towards zero.

## Appendix B. The Fundamentals of Residual Block

The residual block [32] consists of two standard convolution layers and a shortcut connection, which is shown in Figure A1. According to whether the input ($x_{in}$) and the output $\left(y_{\mathrm{out}}\right.$ or $\left.y_{\mathrm{out}}^{*}\right)$ have the same number of channels, the residual block can be divided into two types: (1) the ‘solid line’ shortcut connection, and (2) the ‘dashed line’ shortcut connection.

As for the residual block with the ‘solid line’ shortcut connection (see Figure A1a), the relationship between the input and the output can be expressed by Equation (A4). After two layers of convolution operation, the $\left(x_{\mathrm{in}}\right.$ and $\left.y_{\mathrm{out}}\right)$ have different numbers of channels:

(A4) $$y_{\mathrm{out}}=F\left(x_{\mathrm{in}}\right)+x_{\mathrm{in}}$$

where F(·) refers to the two-layer standard convolution operation, with the padding=1 and stride=1.

As for the residual block with the ‘dashed line’ shortcut connection (see Figure A1b), the relationship between the input $x_{in}$ and the output $y_{\mathrm{out}}^{*}$ can be expressed by Equation (A5). After two layers of convolution operation, $\left(x_{\mathrm{in}}\right.$ and $\left.y_{\mathrm{out}}^{*}\right)$ have different numbers of channels:

(A5) $$y_{\mathrm{out}}^{*}=F^{\prime }\left(x_{\mathrm{in}}\right)+x_{\mathrm{in}}^{*}$$

(A6) $$x_{\mathrm{in}}^{*}=Conv_{-}0p_{-}2s\left(x_{\mathrm{in}},f^{c_{\mathrm{out}}^{2}\times 1\times 1\times c_{\mathrm{in}}^{1}}\right)$$

where $F^{\prime }\left(\cdot \right)$ refers to the two-layer standard convolution operation, with the padding=1. The stride of the first and second convolution layers of $F^{\prime }\left(\cdot \right)$ is 2 and 1, respectively. $Conv_{-}0p_{-}2s\left(\cdot \right)$ refers to the standard convolution function with the padding=0 and stride=2, $f^{c_{\mathrm{out}}^{2}\times 1\times 1\times c_{\mathrm{in}}^{1}}$ refers to the $c_{\mathrm{out}}^{2}$ convolution kernels with the size of $1\times 1\times c_{\mathrm{in}}^{1}$, $c_{\mathrm{in}}^{1}$ is the number of input channels in the first convolution layer of $F^{\prime }\left(\cdot \right)$, and $c_{\mathrm{out}}^{2}$ is the number of output channels in the second convolution layer of $F^{\prime }\left(\cdot \right)$.

## Appendix C. The Fundamentals of GM

The Ghost module (GM) [34] consists of three steps as shown in Figure A2. Firstly, $m\left(m\ll c_{\mathrm{out}}\right)$ convolution kernels are used to generate partial convolution features, denoted as $X^{*}$. Secondly, the new deep linear features $X^{**}$ are generated based on $X^{*}$ by utilizing deep group convolution. Finally, $X^{*}$ and $X^{**}$ are simply spliced together to obtain the final output, which has the same size as the standard convolution output.

Supposing that *X* with the size of $h\times w\times c_{\mathrm{in}}$ is the input data or image of GM, the feature map $X^{*}$ can be expressed by

(A7) $$X^{*}=\mathrm{Conv}\left(X,f^{m\times k\times k\times c_{in}}\right)$$

where Conv(·) refers to the standard convolution function, $f^{m\times k\times k\times c_{in}}$ refers to the $m\left(m\ll c_{\mathrm{out}}\right)$ convolution kernels with the size of $k\times k\times c_{\mathrm{in}}$, and $X^{*}$ is the output with the size of $h_{1}\times w_{1}\times m$.

Based on $X^{*}$, the new linear features $X^{**}$ are generated by utilizing deep group convolution, which can be expressed by

(A8) $$X^{**}=\mathrm{splicing}\left(\varphi_{i}^{i,j}\left(X^{*}\right)\right)\;\forall i=1,\cdots ,m,\;\forall j=1,\cdots ,n-1.$$

where $\varphi_{i}^{i,j}\left(\cdot \right)$ refers to the convolution operation between the *i*th feature map of $X^{*}$ and the *j*th channel of the *i*th group convolution kernel. The size of each group convolution kernel is d×d×(n−1). $X^{**}$ is the output with the size of $h_{1}\times w_{1}\times m\cdot \left(n-1\right)$.

The output *Y* of GM is obtained by simply splicing $X^{*}$ and $X^{**}$ together, which can be expressed by

(A9) $$Y=\mathrm{splicing}\left(X^{*},X^{**}\right)$$

where *Y* is the output with the size of $h_{1}\times w_{1}\times c_{\mathrm{out}}$, $c_{\mathrm{out}}=m\cdot n$.

The ratio of the number for the channels of $X^{*}$ and *Y* is defined as the ‘mapping ratio’, λ=1/n. The number of channels in the feature maps $X^{*}$ and $X^{**}$ is $m=c_{\mathrm{out}}/n$ and $m\cdot \left(n-1\right)=\left(n-1\right)\times c_{\mathrm{out}}/n$, respectively.

## Appendix D. The Fundamentals of CBAM

The convolutional block attention module (CBAM) [35] consists of two steps as shown in Figure A3. CBAM firstly adjust the weight of the channel dimension of the feature map through the channel attention module, and then adjust the weight of spatial dimension of the feature map through the spatial attention module.

Supposing that *X* with the size of $h\times w\times c_{\mathrm{in}}$ is the input data or image of CBAM, the output $X_{1}$ of the channel attention module can be expressed by

(A10) $$X_{1}=X⊗Channel\_W$$

(A11) $$G_{-}{AvgPool}^{c}\_1=Conv\_0p\_0s\left({AvgPool}^{c}\left(X\right),f^{c_{middle}\times 1\times 1\times c_{\mathrm{in}}}\right)$$

(A12) $$G_{-}{AvgPool}^{c}\_2=Conv\_0p\_0s\left({G\_AvgPool}^{c}\_1,f^{c_{in}\times 1\times 1\times c_{\mathrm{middle}}}\right)$$

(A13) $$G_{-}{AvgPool}^{c}\_3=\sigma \left(G_{-}{AvgPool}^{c}\_2\right)$$

(A14) $$G\_{MaxPool}^{c}\_1=Conv\_0p_{-}0s\left({\mathrm{MaxPool}}^{c}\left(X\right),f^{c_{\mathrm{middle}}\times 1\times 1\times c_{in}}\right)$$

(A15) $$G\_{MaxPool}^{c}\_2=Conv\_0p\_0s\left(G\_{MaxPool}^{c}\_1,f^{c_{\mathrm{in}}\times 1\times 1\times c_{\mathrm{middle}}}\right)$$

(A16) $$G\_{MaxPool}^{c}\_3=\sigma \left(G\_{MaxPool}^{c}\_2\right)$$

(A17) $$Channel\_W=G_{-}{AvgPool}^{c}\_3⊕G\_{MaxPool}^{c}\_3$$

where ⊗ refers to the multiplication operation element by element, and Channel_W is the weight of the channel dimension, with the size of $1\times 1\times c_{\mathrm{in}}$. ⊕ refers to the addition operation element by element. ${AvgPool}^{c}$ and ${MaxPool}^{c}$ refer to the avg-pooling and max-pooling along the direction of the channel dimension, respectively. Conv_0p_0s(·) refers to the standard convolution function with the padding=0 and stride=0. *G* refers to a two-layer convolution operation with the second convolution layer activated by a sigmoid function. The first convolution layer is carried out based on the convolution kernels with the size of $1\times 1\times c_{\mathrm{in}}$, the second convolution layer is carried out based on the $c_{\mathrm{in}}$ convolution kernels with the size of $1\times 1\times c_{\mathrm{middle}}$. σ refers to the sigmoid function.

For the two convolution kernels in $G_{-}{AvgPool}^{c}$ and $G\_{MaxPool}^{c}$, $r=c_{in}/c_{\mathrm{middle}}$ is defined as the ‘compression ratio’ of the channel attention module, r>1.

Next, the output $X_{1}$ is sent to the spatial attention module. The output $X_{2}$ of CBAM can be expressed by

(A18) $$X_{2}=X_{1}⊗Spatial\_W$$

(A19) $$\mathrm{Spatial}\_W=\sigma ({\mathrm{Conv}}_{3p\_1s}(\left[{\mathrm{AvgPool}}^{s}\left(X_{1}\right),{\mathrm{MaxPool}}^{s}\left(X_{1}\right)\right],f^{\left(1\times 7\times 7\times 2\right)}))$$

where ⊕ refers to the multiplication operation element by element, Spatial_W is the weight of the spatial dimension (along the height and width direction), with the size of h×w×1. ${\mathrm{AvgPool}}^{s}$ and ${\mathrm{MaxPool}}^{s}$ refer to the avg-pooling and max-pooling along the direction of the spatial dimension, respectively. Conv_3p_1s(·) refers to the standard convolution function with the padding=3 and stride=1, and $f^{\left(1\times 7\times 7\times 2\right)}$ refers to the convolution kernel with the size of 7×7×2. σ refers to the sigmoid function.

## Appendix E. The Fundamentals of Grad-CAM

The Gradient-weighted Class Activation Mapping (Grad-CAM) was proposed by Selvaraju et al. [38], which can be utilized to carry out interpretable analysis of the ‘black box’ model. This technique can be integrated into any deep learning model without changing the original structure, which ensures the reliability of the interpretable analysis.

The implementation of Grad-CAM is shown as in Figure A4. It consists of three major steps: forward propagation, backward propagation, post-processing. It is introduced as follows.

In forward propagation, the original ECG image (RGB) is used as input. Then, the feature map is obtained by extracting features through convolutional layers. The score of each category can be obtained through the following fully connected layer.

In backward propagation, the partial derivatives $A^{\left\{class\right\}}$ of the score $y^{\left\{class\right\}}$ on feature map are obtained. Then, the weight matrix $W^{\left\{class\right\}}$ can be obtained by the avg-pooling operation along the channel direction of $A^{\left\{class\right\}}$, which is given by Equation (A21). Before post-processing, the feature map ‘class-discriminative localization map’ $L_{\mathrm{Gard}-\mathrm{CAM}}^{\mathrm{class}}$ needs to be calculated according to Equation (A22):

(A20) $$A^{\left\{\mathrm{class}\right\}}=\frac{\partial y^{\mathrm{class}}}{\partial A}$$

(A21) $$W^{class}=AvgPool^{c}\left(A^{\left\{class\right\}}\right)$$

(A22) $$L_{\mathrm{Gard}-\mathrm{CAM}}^{\mathrm{class}}=\mathrm{Relu}\left({\mathrm{Sum}}^{s}\left(W^{\mathrm{class}}⊗A\right)\right)$$

where $AvgPool^{c}$ refers to avg-pooling along the direction of the channel dimension, ⊕ refers to the multiplication operation element by element, and ${\mathrm{Sum}}^{c}$ refers to the sum operation along the direction of the spatial dimension.

In post-processing, visualization processing is carried out. Firstly, the elements of the original ECG and $L_{\mathrm{Gard}-\mathrm{CAM}}^{\mathrm{class}}$ are scaled to [0,1]. Secondly, $L_{\mathrm{Gard}-\mathrm{CAM}}^{\mathrm{class}}$ is reshaped to the same size of the original ECG by linear interpolation and then converted to a three-channel pseudo color image $PCI\_L_{\mathrm{Gard}-\mathrm{CAM}}^{\mathrm{class}}$ by the Jet color mapping algorithm [49]. Finally, the colorful ECG thermal map can be obtained by remapping the new feature map, which is obtained by adding the original ECG and the pseudo color image $PCI\_L_{\mathrm{Gard}-\mathrm{CAM}}^{\mathrm{class}}$, dividing each element by the maximum value of the corresponding channel, and then multiplying by 255.

## Appendix F. Network Structure

**Table A1:** The network structure of ResNet19.

Module	Layer	Filter	WinSize	[p s]	Input	Output	Num_params
	Conv1	[16 3 3 3]		[1 1]	[224 224 3]	[224 224 16]	432
	Max-pooling		[3 3]	[1 2]	[224 224 16]	[112 112 16]	
Residual_solid	Conv2	[16 3 3 16]		[1 1]	[112 112 16]	[112 112 16]	2304
	Conv3	[16 3 3 16]		[1 1]	[112 112 16]	[112 112 16]	2304
Residual_solid	Conv4	[16 3 3 16]		[1 1]	[112 112 16]	[112 112 16]	2304
	Conv5	[16 3 3 16]		[1 1]	[112 112 16]	[112 112 16]	2304
Residual_solid	Conv6	[16 3 3 16]		[1 1]	[112 112 16]	[112 112 16]	2304
	Conv7	[16 3 3 16]		[1 1]	[112 112 16]	[112 112 16]	2304
Residual_dashed	Conv8	[32 3 3 16]		[1 2]	[112 112 16]	[56 56 32]	4608
	Conv9	[32 3 3 32]		[1 1]	[56 56 32]	[56 56 32]	9216
	ShortCutConv1	[32 1 1 16]		[0 2]	[112 112 16]	[56 56 32]	512
Residual_solid	Conv10	[32 3 3 32]		[1 1]	[56 56 32]	[56 56 32]	9216
	Conv11	[32 3 3 32]		[1 1]	[56 56 32]	[56 56 32]	9216
Residual_solid	Conv12	[32 3 3 32]		[1 1]	[56 56 32]	[56 56 32]	9216
	Conv13	[32 3 3 32]		[1 1]	[56 56 32]	[56 56 32]	9216
Residual_dashed	Conv14	[64 3 3 32]		[1 2]	[56 56 32]	[28 28 64]	18,432
	Conv15	[64 3 3 64]		[1 1]	[28 28 64]	[28 28 64]	36,864
	ShortCutConv2	[64 1 1 32]		[0 2]	[56 56 32]	[28 28 64]	2048
Residual_solid	Conv16	[64 3 3 64]		[1 1]	[28 28 64]	[28 28 64]	36,864
	Conv17	[64 3 3 64]		[1 1]	[28 28 64]	[28 28 64]	36,864
Residual_solid	Conv18	[64 3 3 64]		[1 1]	[28 28 64]	[28 28 64]	36,864
	Conv19	[64 3 3 64]		[1 1]	[28 28 64]	[28 28 64]	36,864
	Avg-pooling		[28 28]	[0 0]	[28 28 64]	[1 1 64]	
	Fully connected	[3 1 1 64]			[1 1 64]	[1 1 3]	192
Total							270,448

Note: ‘Residual_solid’ and ‘Residual_dashed’ refer to the residual blocks with a ‘solid line’ and ‘dashed line’, respectively, as illustrated in Figure A1. ‘Conv’ is the abbreviation of convolution. ‘ShortCutConv’ refers to the convolution operation on the dashed line of ‘Residual_dashed’. ‘Filter’ refers to the convolution kernels $f^{c_{\mathrm{out}}\times k\times k\times c_{\mathrm{in}}}$, and its size is expressed as $\left[C_{\mathrm{out}},k,k,C_{\mathrm{in}}\right]$. ‘WinSize’ refers to the sliding window size in max-pooling and avg-pooling. ‘[p s]’ refers to the [padding,stride] in the convolution and pooling operations. ‘Num_params’ refers to the number of model parameters in each layer.

**Table A2:** The network structure of GM-ResNet19.

Module	Layer	Filter1	Filter2	WinSize	[p s]	Num_params
	GM1	[8 3 3 3]	[3 3 1] × 8		[1 1]	288
	Max-pooling			[3 3]	[1 2]	
Residual_solid	GM2	[8 3 3 16]	[3 3 1] × 8		[1 1]	1224
	GM3	[8 3 3 16]	[3 3 1] × 8		[1 1]	1224
Residual_solid	GM4	[8 3 3 16]	[3 3 1] × 8		[1 1]	1224
	GM5	[8 3 3 16]	[3 3 1] × 8		[1 1]	1224
Residual_solid	GM6	[8 3 3 16]	[3 3 1] × 8		[1 1]	1224
	GM7	[8 3 3 16]	[3 3 1] × 8		[1 1]	1224
Residual_dashed	GM8	[16 3 3 16]	[3 3 1] × 16		[1 2]–[1 1]	2448
	GM9	[16 3 3 32]	[3 3 1] × 16		[1 1]	4752
	ShortCutGM1	[16 1 1 16]	[3 3 1] × 16		[0 2]–[1 1]	400
Residual_solid	GM10	[16 3 3 32]	[3 3 1] × 16		[1 1]	4752
	GM11	[16 3 3 32]	[3 3 1] × 16		[1 1]	4752
Residual_solid	GM12	[16 3 3 32]	[3 3 1] × 16		[1 1]	4752
	GM13	[16 3 3 32]	[3 3 1] × 16		[1 1]	4752
Residual_dashed	GM14	[32 3 3 32]	[3 3 1] × 32		[1 2]–[1 1]	9504
	GM15	[32 3 3 64]	[3 3 1] × 32		[1 1]	18,720
	ShortCutGM2	[32 1 1 32]	[3 3 1] × 32		[0 2]–[1 1]	1312
Residual_solid	GM16	[32 3 3 64]	[3 3 1] × 32		[1 1]	18,720
	GM17	[32 3 3 64]	[3 3 1] × 32		[1 1]	18,720
Residual_solid	GM18	[32 3 3 64]	[3 3 1] × 32		[1 1]	18,720
	GM19	[32 3 3 64]	[3 3 1] × 32		[1 1]	18,720
	Avg-pooling			[28 28]	[0 0]	
	Fully connected	[3 1 1 64]				192
Total						138,848

Note: ‘Residual_solid’, ‘Residual_dashed’, ‘WinSize’, ‘[p s]’, and ‘Num_params’ are provided in Table A1. ‘GM’ refers to the Ghost module. ‘ShortCutGM’ refers to the GM operation on the dashed line of ‘Residual_dashed’. ‘Filter1’ and ‘Filter2’ refer to the standard convolution kernels $f^{m\times k\times k\times c_{\mathrm{in}}}$ and the group convolution kernels $m\times f^{d\times d\times \left(n-1\right)}$ in GM, respectively. The sizes of the input and output in each residual block are the same as those in ResNet19.

**Table A3:** The network structure of CBAM-ResNet19.

Module	Layer	Filter1	Filter2	Filter3	WinSize	[p s]	Num_params
	Conv1	[16 3 3 3]				[1 1]	432
	Max-pooling				[3 3]	[1 2]	
Residual_solid	Conv2	[16 3 3 16]				[1 1]	2304
	Conv3	[16 3 3 16]				[1 1]	2304
	CBAM	[2 1 1 16]	[16 1 1 2]	[1 7 7 2]		[0 0]–[0 0]–[3 1]	162
Residual_solid	Conv4	[16 3 3 16]				[1 1]	2304
	Conv5	[16 3 3 16]				[1 1]	2304
	CBAM	[2 1 1 16] × 2	[16 1 1 2] × 2	[1 7 7 2]		[0 0]–[0 0]–[3 1]	162
Residual_solid	Conv6	[16 3 3 16]				[1 1]	2304
	Conv7	[16 3 3 16]				[1 1]	2304
	CBAM	[2 1 1 16] × 2	[16 1 1 2] × 2	[1 7 7 2]		[0 0]–[0 0]–[3 1]	162
Residual_dashed	Conv8	[32 3 3 16]				[1 2]–[1 1]	4608
	Conv9	[32 3 3 32]				[1 1]	9216
	CBAM	[4 1 1 32] × 2	[32 1 1 4] × 2	[1 7 7 2]		[0 0]–[0 0]–[3 1]	354
	ShortCutCov1	[32 1 1 16]				[0 2]	512
Residual_solid	Conv10	[32 3 3 32]				[1 1]	9216
	Conv11	[32 3 3 32]				[1 1]	9216
	CBAM	[4 1 1 32] × 2	[32 1 1 4] × 2	[1 7 7 2]		[0 0]–[0 0]–[3 1]	354
Residual_solid	Conv12	[32 3 3 32]				[1 1]	9216
	Conv13	[32 3 3 32]				[1 1]	9216
	CBAM	[4 1 1 32] × 2	[32 1 1 4] × 2	[1 7 7 2]		[0 0]–[0 0]–[3 1]	354
Residual_dashed	Conv14	[64 3 3 32]				[1 2]–[1 1]	18,432
	Conv15	[64 3 3 64]				[1 1]	36,864
	CBAM	[8 1 1 64] × 2	[64 1 1 8] × 2	[1 7 7 2]		[0 0]–[0 0]–[3 1]	1122
	ShortCutCov2	[64 1 1 32]				[0 2]	2048
Residual_solid	Conv16	[64 3 3 64]				[1 1]	36,864
	Conv17	[64 3 3 64]				[1 1]	36,864
	CBAM	[8 1 1 64] × 2	[64 1 1 8] × 2	[1 7 7 2]		[0 0]–[0 0]–[3 1]	1122
Residual_solid	Conv18	[64 3 3 64]				[1 1]	36,864
	Conv19	[64 3 3 64]				[1 1]	36,864
	CBAM	[8 1 1 64] × 2	[64 1 1 8] × 2	[1 7 7 2]		[0 0]–[0 0]–[3 1]	1122
	Avg-pooling				[28 28]	[0 0]	
	Fully connected	[3 1 1 64]					192
Total							275,362

Note: ‘Residual_solid’, ‘Residual_dashed’, ‘WinSize’, ‘[p s]’, ‘Num_params’, ‘Conv’, and ‘ShortCutConv’ are provided in Table A1. ‘CBAM’ refers to the convolutional block attention module. As for ‘CBAM’, ‘Filter1’ and ‘Filter2’ are used in the channel attention module, while ‘Filter3’ is used in the spatial attention module. The sizes of the input and output in each residual block are the same as those in ResNet19.

**Table A4:** The network structure of GM-CBAM-ResNet19.

Module	Layer	Filter1	Filter2	Filter3	WinSize	[p s]	Num_params
	GM1	[8 3 3 3]	[3 3 1] × 8			[1 1]	288
	Max-pooling				[3 3]	[1 2]	
Residual_solid	GM2	[8 3 3 16]	[3 3 1] × 8			[1 1]	1224
	GM3	[8 3 3 16]	[3 3 1] × 8			[1 1]	1224
	CBAM	[2 1 1 16] × 2	[16 1 1 2] × 2	[1 7 7 2]		[0 0]–[0 0]–[3 1]	226
Residual_solid	GM4	[8 3 3 16]	[3 3 1] × 8			[1 1]	1224
	GM5	[8 3 3 16]	[3 3 1] × 8			[1 1]	1224
	CBAM	[2 1 1 16] × 2	[16 1 1 2] × 2	[1 7 7 2]		[0 0]–[0 0]–[3 1]	226
Residual_solid	GM6	[8 3 3 16]	[3 3 1] × 8			[1 1]	1224
	GM7	[8 3 3 16]	[3 3 1] × 8			[1 1]	1224
	CBAM	[2 1 1 16] × 2	[16 1 1 2] × 2	[1 7 7 2]		[0 0]–[0 0]–[3 1]	226
Residual_dashed	GM8	[16 3 3 16]	[3 3 1] × 16			[1 2]–[1 1]	2448
	GM9	[16 3 3 32]	[3 3 1] × 16			[1 1]	4752
	CBAM	[4 1 1 32] × 2	[32 1 1 4] × 2	[1 7 7 2]		[0 0]–[0 0]–[3 1]	610
	ShortCutGM1	[16 1 1 16]	[3 3 1] × 16			[0 2]–[1 1]	400
Residual_solid	GM10	[16 3 3 32]	[3 3 1] × 16			[1 1]	4752
	GM11	[16 3 3 32]	[3 3 1] × 16			[1 1]	4752
	CBAM	[4 1 1 32] × 2	[32 1 1 4] × 2	[1 7 7 2]		[0 0]–[0 0]–[3 1]	610
Residual_solid	GM12	[16 3 3 32]	[3 3 1] × 16			[1 1]	4752
	GM13	[16 3 3 32]	[3 3 1] × 16			[1 1]	4752
	CBAM	[4 1 1 32] × 2	[32 1 1 4] × 2	[1 7 7 2]		[0 0]–[0 0]–[3 1]	610
Residual_dashed	GM14	[32 3 3 32]	[3 3 1] × 32			[1 2]–[1 1]	9504
	GM15	[32 3 3 64]	[3 3 1] × 32			[1 1]	18,720
	CBAM	[8 1 1 64] × 2	[64 1 1 8] × 2	[1 7 7 2]		[0 0]–[0 0]–[3 1]	2146
	ShortCutGM2	[32 1 1 32]	[3 3 1] × 32			[0 2]–[1 1]	1312
Residual_solid	GM16	[32 3 3 64]	[3 3 1] × 32			[1 1]	18,720
	GM17	[32 3 3 64]	[3 3 1] × 32			[1 1]	18,720
	CBAM	[8 1 1 64] × 2	[64 1 1 8] × 2	[1 7 7 2]		[0 0]–[0 0]–[3 1]	2146
	Avg-pooling				[28 28]	[0 0]	
	Fully connected	[3 1 1 64]					192
Total							147,794

Note: ‘Residual_solid’, ‘Residual_dashed’, ‘WinSize’, ‘[p s]’ and ‘Num_params’ are provided in Table A1. ‘GM’ and ‘ShortCutGM’ are provided in Table A2. ‘CBAM’ is provided in Table A3. ‘GM’ contains two types of filter (i.e., convolution kernel). ‘CBAM’ contains three categories of filter (i.e., convolution kernel). The sizes of the input and output in each residual block are the same as those in ResNet19 and GM-ResNet19.
